# Pauperism in England in 1857

**Published:** 1857-04

**Authors:** 


### PAUPERISM IN ENGLAND IN 1857.

The number of paupers of all classes in England and Wales, in receipt of relief, on the 1st of January, 1857, was 843,430; the whole population, according to the census of 1851, being 16,530,262. The poor thus relieved were distributed through 624 unions, and 14,169 parishes. After deducting increase, the total relieved leaves a decrease of 33,225 relieved on the same date in the year 1856, *i.e.*, a decrease of 3·8 per cent., and affords a proportion of all classes relieved of 5·1 per cent. to the whole population. Of the classes thus relieved, 139,130, or 16·5 per cent., rank as able bodied paupers, yielding a decrease of 13,034, or 8·6 per cent., from the year preceding. Of these able bodied persons, 22,368 received in-door, and 116,762 (amongst whom 50,362 were widows) out-door relief. This calculation does not include places under "Gilbert's Act," under "local Acts," and the parishes under "Elizabeth's Act." In these there are collectively comprised 435 parishes, and a population of 1,371,409, regarding which no particulars are supplied. (*Government Return, February,* 1857.)

